# CD1d-antibody fusion proteins target iNKT cells to the tumor and trigger long-term therapeutic responses

**DOI:** 10.1007/s00262-012-1381-7

**Published:** 2012-12-15

**Authors:** Stéphanie Corgnac, Rachel Perret, Laurent Derré, Lianjun Zhang, Kathrin Stirnemann, Maurice Zauderer, Daniel E. Speiser, Jean-Pierre Mach, Pedro Romero, Alena Donda

**Affiliations:** 1 Ludwig Center for Cancer Research, University of Lausanne, 1066 Epalinges, Switzerland; 2 Urology Research Unit, Department of Urology, Lausanne University Hospital, 1005 Lausanne, Switzerland; 3 Roche Pharma AG, 4153 Reinach, Switzerland; 4 Vaccinex Inc., Rochester, NY USA; 5 Department of Biochemistry, University of Lausanne, 1066 Epalinges, Switzerland

**Keywords:** Cancer immunotherapy, iNKT cells, CD1d, Tumor targeting, Fusion protein

## Abstract

Despite the well-established antitumor activity of CD1d-restricted invariant natural killer T lymphocytes (iNKT), their use for cancer therapy has remained challenging. This appears to be due to their strong but short-lived activation followed by long-term anergy after a single administration of the CD1d agonist ligand alpha-galactosylceramide (αGC). As a promising alternative, we obtained sustained mouse iNKT cell responses associated with prolonged antitumor effects through repeated administrations of tumor-targeted recombinant sCD1d-antitumor scFv fusion proteins loaded with αGC. Here, we demonstrate that CD1d fusion proteins bound to tumor cells via the antibody fragment specific for a tumor-associated antigen, efficiently activate human iNKT cell lines leading to potent tumor cell lysis. The importance of CD1d tumor targeting was confirmed in tumor-bearing mice in which only the specific tumor-targeted CD1d fusion protein resulted in tumor inhibition of well-established aggressive tumor grafts. The therapeutic efficacy correlated with the repeated activation of iNKT and natural killer cells marked by their release of TH1 cytokines, despite the up-regulation of the co-inhibitory receptor PD-1. Our results demonstrate the superiority of providing the superagonist αGC loaded on recombinant CD1d proteins and support the use of αGC/sCD1d-antitumor fusion proteins to secure a sustained human and mouse iNKT cell activation, while targeting their cytotoxic activity and cytokine release to the tumor site.

**Electronic supplementary material:**

The online version of this article (doi:10.1007/s00262-012-1381-7) contains supplementary material, which is available to authorized users.

## Introduction

Human Vα24-invariant natural killer T lymphocytes (iNKT), and their murine counterparts Vα14-iNKT cells, represent a particular sublineage of T lymphocytes activated by self- and microbial-derived glycolipids in the context of the monomorphic MHC-related molecule CD1d. Their importance in the transactivation of innate and adaptive immune responses has been extensively described [1, 2], as well as their protective or pathological role in various conditions [3]. In particular, their antitumor activity has been well documented in a number of mouse tumor models [4–6], and several clinical observations also indicate their protective role against cancer progression. Furthermore, low numbers and impaired proliferative capacity of iNKT cells were reported in cancer patients compared to normal donors [7, 8], which in some studies were correlated with poor clinical outcome [9, 10]. These preclinical and clinical observations have prompted testing of iNKT cell-directed therapies, mainly through their strong activation by the synthetic glycosphingolipid CD1d ligand, alpha-galactosylceramide (αGC) [11–13]. Phase I clinical trials involving the autologous transfer of αGC-pulsed monocyte-derived dendritic cells (moDC) were conducted in patients with different types of cancer [4, 11, 13–16]. No severe adverse effects were seen and the transient expansion and activation of iNKT cells, obtained in non-small-cell lung cancer (NSCLC) and head and neck squamous cell cancer (HNSCC) patients, correlated with some clinical benefit. Unfortunately, however, iNKT cell-mediated tumor immunotherapy has been limited by the short-lived cytokine response of iNKT cells to αGC stimulation, followed by a long-term anergy [4, 17, 18]. Recently, we have instead showed that sustained mouse iNKT cell responses could be induced by repeated stimulations with recombinant αGC-loaded sCD1d fusion proteins [19]. This prolonged responsiveness of iNKT cells resulted in potent antitumor activity when CD1d was targeted to the tumor site by its fusion to an anti-HER2 antitumor antibody fragment [19]. In the present study, recombinant CD1d proteins are shown to expand and activate human iNKT cells without the need of antigen-presenting cells (APCs). Importantly, we show that human iNKT cells exhibit a potent direct cytotoxicity only against cancer cells coated with the specific sCD1d-antitumor scFv fusion protein. The importance of CD1d tumor targeting to promote sustained activation of iNKT cells and prolonged tumor inhibition is further characterized in mice in therapeutic settings.

## Materials and methods

### Mice and human samples

Female mice C57BL/6J (B6) 6–8 weeks old (Harlan, Zeist, Holland) were maintained in specific pathogen-free conditions. All animal experiments were conducted according to institutional guidelines and under an authorization delivered by the Swiss veterinary department. Fresh human PBMC were obtained from healthy donor blood, isolated by density centrifugation using Lymphoprep (Axis-Shield PoC AS, Norway).

### Tumor cell lines and human iNKT cell lines and clones

The murine colon carcinoma MC-38 cell line transfected with human CEA (MC38-CEA) was a kind gift from J. Primus [20]. The human cell lines KATO III (gastric carcinoma) and SK-BR-3 (breast carcinoma) were obtained from the ATCC. The human B lymphoma cell line C1R stably transfected with human CD1d was used as APC. Alternatively, moDCs were generated as described by Shao et al. [21]. Human iNKT cell lines were established starting with fresh PBMC from healthy donors cultured with αGC (100 ng/ml) or αGC/sCD1d proteins (40 μg/ml) in RPMI medium with 8 % human serum, recombinant IL-2 (20 U/ml) and IL-7 (10 ng/ml). Human iNKT cell clones had been previously generated by limiting dilution after sorting from peripheral blood lymphocytes (PBLs) of healthy donors by anti-CD3, anti-Vα24, and anti-Vβ11 mAbs staining [22].

### Reagents and antibodies

The αGC analog KRN7000 (Alexis Biochemicals Corp) was dissolved in PBS-0.5 % Tween-20. Cytokine levels were measured either individually by ELISA (ELISA ready-set-go, eBiosciences), or as multiple cytokine measurements using BD Cytometric Bead Array kit TH1/TH2/TH17 (CBA, BD Biosciences). All fluorochrome-labeled antibodies were purchased from Becton–Dickinson (BD Biosciences) or eBiosciences. The humanized mAbs anti-HER2 Herceptin (Trastuzumab) was from Roche Ltd and anti-CEA (X4) from Ciba-Geigy [23]. Cells were analyzed with a FACSCalibur, FACSCanto or LSRII (BD Biosciences) and the acquired data were processed using FlowJo software (Tree Star Inc.).

### Recombinant CD1d fusion proteins

Genetic fusion of mouse β2 microglobulin (β2 m) with the soluble part of mouse CD1d (sCD1d) has been described previously [19]. In the original pEAK8-β2m-sCD1d-anti-HER2-6xHIS construct, the anti-HER2 scFv located between the Gly-Ser spacer and the 6xHIS was replaced by the anti-CEA scFv MFE23 (kindly provided by R.H. Begent [24]). Recombinant CD1d fusion proteins produced by transient transfection of the human cell line HEK293-EBNA (Cellular Biotechnology Laboratory, EPFL, Switzerland) were purified and loaded with αGC as previously reported [19] (Fig. S1). The CD1d tetramer was developed by engineering a BirA consensus sequence at the C-terminus of the soluble mouse CD1d protein. The CD1d monomer was biotinylated by the BirA enzyme (Avidity, Denver, CO), and after loading with αGC, it was tetramerized on streptavidin-PE (Invitrogen) using a molar ratio of 4:1.

### In vitro proliferation

Human iNKT cells were labeled with 1 μM CFSE for 6 min at 37 °C and washed three times. Labeled iNKT cells were incubated in a 12-well plate at 1 × 10^6^ cells/ml in RPMI with 8 % HS, 30 U/ml IL-2, and 10 ng/ml IL-7 at 37 °C. iNKT cells were stimulated with either 8 × 10^5^ irradiated αGC-loaded C1R-CD1d, plastic-coated sCD1d fusion proteins (40 μg), or 200 ng/ml αGC. The dilution of CFSE was analyzed by flow cytometry.

### iNKT cell cytotoxicity

For chromium release experiments, iNKT clones, CD4^+^ or double negative (DN), were thawed the day before and kept overnight in RPMI 8 %HS supplemented with recombinant IL-2 (150 U/ml) and IL-7 (10 ng/ml). On the day of the experiment, target cells were labeled with ^51^Cr for 1 h at 37 °C and washed three times in medium before incubation (10^3^ cells per well) in 96 V-bottom well plates either with effector cells at an E:T ratio of 10:1 and different concentration of CD1d-recombinant molecules, or in the presence of different E:T ratios and a fixed concentration of CD1d-recombinant molecules (10 μg/ml). Supernatants were collected after 4 h of incubation at 37 °C, and released radioactivity was measured in a γ-counter. For Annexin V analysis, 2 × 10^5^ NKT cells were incubated with 1 × 10^4^ tumor cells in the presence of either αGC/sCD1d-antitumor scFv at 10 μg/ml in RPMI 8 % human serum or 5 × 10^4^ αGC/C1R-CD1d cells or 5 × 10^4^ αGC/moDCs for 4 h at 37 °C. After incubation, tumor cells and APCs were analyzed using Annexin V kit (BD Pharmingen) and CD1d-tetramer-positive iNKT cells were stained for CD107a and intracellular content of IFNγ and TNFα.

### Antitumor therapy

Mice were grafted s.c. in the right flank with 7 × 10^5^ MC38-CEA cells. When all tumors were palpable, mice were treated i.v. with 200 μl of either PBS alone, equimolar amounts of αGC (0.4 μg), αGC/sCD1d (25 μg), or αGC/CD1d-antitumor scFv fusion proteins (40 μg). Systemic treatment was repeated at 3- to 4-day intervals. Mean tumor volume measured every 2 days was calculated using the following formula: (length × width × thickness)/2.

### Statistical analysis

Results are expressed as mean ± SEM. Statistical significance between the groups was determined with student’s *t* test or one-way -ANOVA test with Bonferroni correction (GraphPad Prism, GraphPad software). Tumor progression statistics were calculated with two-way ANOVA test with Bonferroni correction (GraphPad Prism, GraphPad software).

## Results

### Human iNKT cells efficiently proliferate in the presence of αGC-loaded CD1d protein

**Fig. 1:**
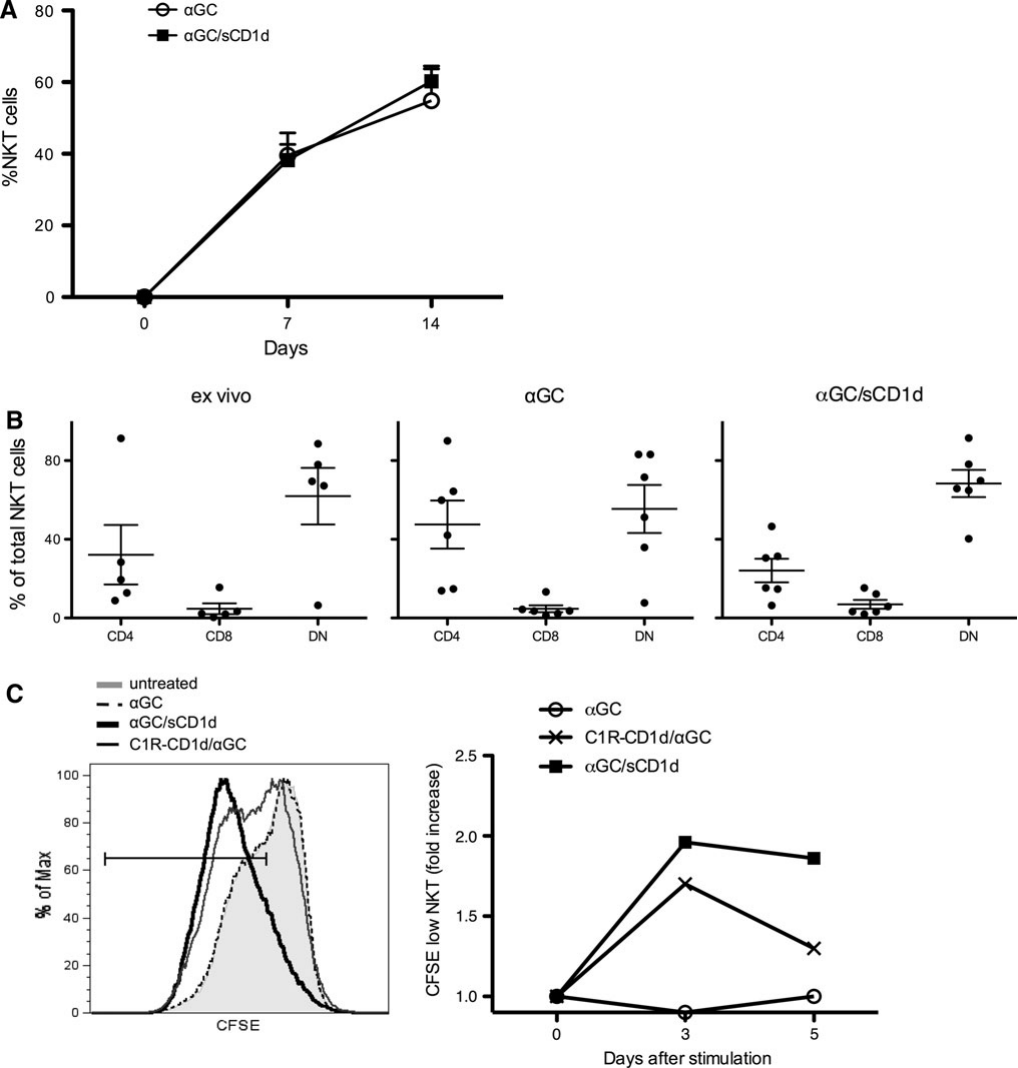
Expansion of human iNKT cell lines by αGC/sCD1d proteins. **a** PBMCs from healthy donors were stimulated with medium alone, αGC (100 ng/ml) or αGC/sCD1d protein (10 μg/ml). Frequency of iNKT cells in total PBMC was assessed as CD1d-tetramer^+^CD3^+^ at day 0, 7, and 14 of culture. Results are shown as mean ± SEM of four donors. **b** Distribution of human iNKT cell subsets ex vivo and after 14 days of culture as described in **a**.* Dots* represent percentages of CD4, CD8, and DN iNKT cells in total CD1d-tetramer^+^CD3^+^ from normal individuals or expanded cell lines, and* bars* show mean ± SEM. **c** CFSE-labeled iNKT cells were incubated for 5 days with the different stimuli, and CFSE dilution was analyzed by FACS on gated CD1d-tetramer^+^CD3^+^ cells.* Left panel* illustrates CFSE fluorescence in iNKT cells on day 5 of culture. *Right panel* shows the kinetic of CFSE dilution as the fold increase of CFSE^low^ iNKT cells. Results shown are representative of three independent experiments

To validate the usefulness of soluble recombinant CD1d proteins for clinical immunotherapy, we investigated the reactivity of human iNKT cells to mouse αGC/sCD1d or αGC/sCD1d-antitumor scFv proteins. Irrespectively of whether fused or not to an antitumor scFv fragment, all sCD1d fusion proteins in solution were able to expand iNKT cell lines from freshly isolated human PBMC. The kinetics of expansion was similar to that observed following exposure to free αGC (Fig. 1a), with approximately 40 % iNKT cells on day 7 and 60 % on day 14 of culture. All iNKT cell lines, whether expanded with free αGC or αGC-loaded sCD1d fusion proteins, retained the same subset composition, with a majority of DN and a minority of CD8^+^ iNKT cells (Fig. 1b). Importantly, recombinant αGC/sCD1d proteins could directly expand pure iNKT cell populations, as seen by CFSE dilution (Fig. 1c) and increased numbers of iNKT cells over 5 days of culture (data not shown), whereas the addition of irradiated APCs was required for free αGC to induce iNKT cell proliferation. These data indicate that αGC-loaded recombinant CD1d proteins directly trigger the semi-invariant TCR of human iNKT cells, and thus represent a promising tool for rapid and potent expansion of human iNKT cells from patients for subsequent adoptive cell transfer.

### Soluble CD1d proteins directly activate human iNKT cell clones without requirement for APCs

**Fig. 2:**
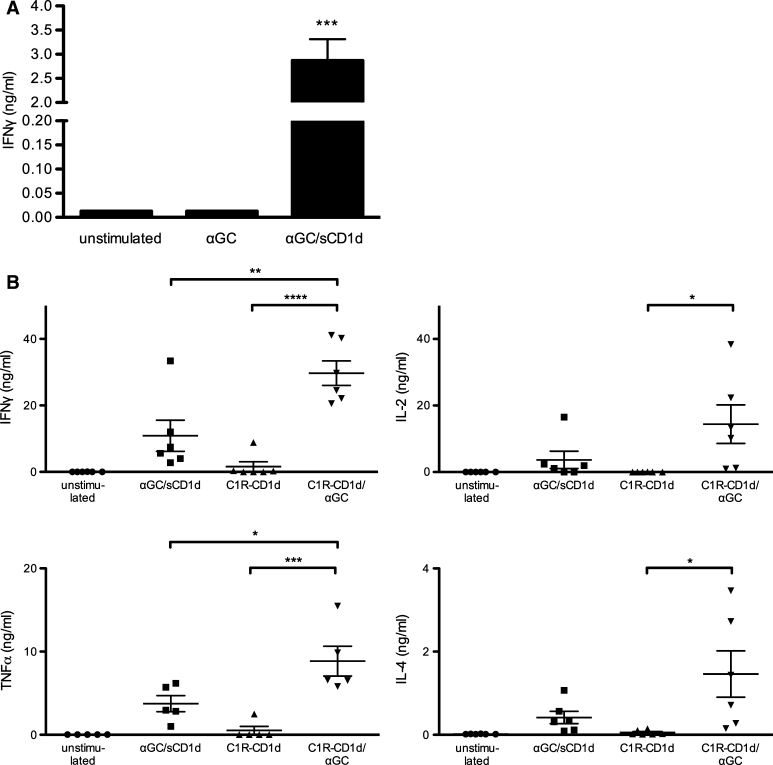
Human iNKT cells are directly activated by recombinant αGC/sCD1d proteins. **a** iNKT cell clones (10^5^) were incubated for 18 h with αGC (100 ng/ml) or αGC/sCD1d proteins in solution (10 μg/ml).* Graph* shows the level of IFNγ in the supernatant as the mean ± SEM of three different human iNKT cell clones. ***P < 0.001. **b** Activation of iNKT cells by αGC/sCD1d proteins or αGC-pulsed APCs. Human iNKT cell clones (10^5^) were incubated with no stimulus, with plastic-coated αGC/sCD1d proteins (10 μg) or with the B-cell lymphoma cell line C1R-CD1d (5 × 10^4^) pulsed or not with αGC. After 24 h, cytokines were measured in supernatants by CBA. *P < 0.01**P < 0.005, ***P < 0.001, ****P < 0.0001

As suggested by the expansion of human iNKT cells, αGC-loaded sCD1d proteins did not require the presence of APCs and were sufficient to activate human iNKT cell clones to release IFNγ after 18-h incubation (Fig. 2a). In contrast, αGC as a free drug was unable to activate iNKT cell clones in the absence of APCs (Fig. 2a) and required the presence of CD1d-expressing cells such as the human lymphoma C1R transfected with CD1d (Fig. 2b). These data fully established that the activation of human iNKT cells by soluble CD1d proteins did not result from the transfer of αGC to endogenously expressed CD1d, but rather from the direct TCR triggering by the soluble fusion proteins. As shown for iNKT cell proliferation, plastic-coated sCD1d proteins were even more efficient than soluble proteins in inducing iNKT cell clones to release a panel of cytokines such as IFNγ, TNFα, IL-2, and IL-4 (Fig. 2b). Still, when compared to αGC loaded on C1R-CD1d APCs, sCD1d proteins remained about threefold weaker in activating iNKT cells, likely resulting from the lack of adhesion mechanisms and molecular aggregation provided by cell–cell interaction.

### Human iNKT cells efficiently kill tumor cells only when coated with the sCD1d-antitumor fusion protein

**Fig. 3:**
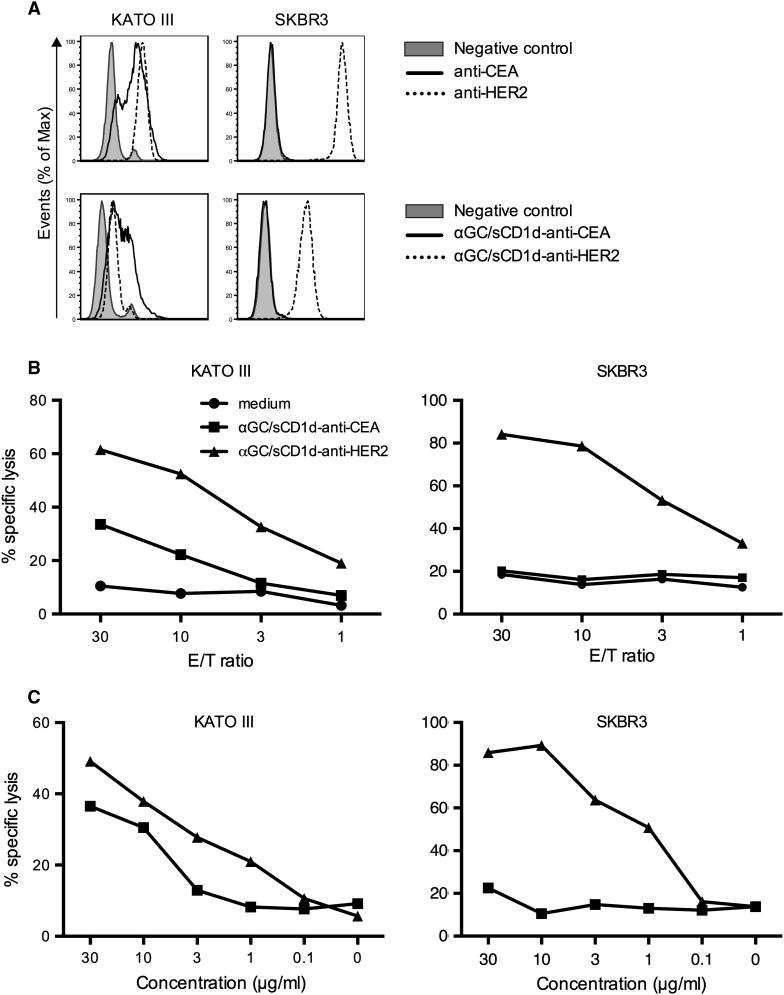
Human iNKT cells efficiently kill tumor cells only when coated with the αGC/sCD1d-antitumor fusion protein. **a** Expression of HER2 and CEA by KATO III and SKBR3 human tumor cells revealed either by the anti-HER2 (Herceptin) and anti-CEA (X4) mAbs (*upper panels*), or by the binding of sCD1d-anti-CEA and sCD1d–anti-HER2 fusion proteins, revealed by FITC-labeled anti-CD1d (*lower panels*). **b** ^51^Cr release assay after 4-h incubation of iNKT cell clones with the tumor cell lines described in **a**.* Graph curves* show percent killing of KATO III (*left panel*) and SKBR-3 cells (*right panel*) with decreasing effector-to-target ratio (*E/T*) with 2 × 10^3^ target tumor cells and 10 μg/ml of sCD1d fusion proteins. **c** *Panels* show percent killing of KATO III (*left*) and SKBR-3 cells (*right*) with decreasing concentrations of sCD1d fusion proteins at an E/T ratio of 10/1. Results shown are representative of three independent experiments

In view of the rare expression of CD1d on tumor cells, the direct cytotoxicity of iNKT cells against tumors has been disregarded, and instead, the immediate antitumor activity of iNKT cells was shown to be largely mediated by the transactivation of natural killer cells [5, 19, 25]. However, direct cytotoxicity of human iNKT cells has been well demonstrated, especially against CD1d-expressing leukemia in vitro and in vivo [26, 27]. Here, we show that the killing capacity of human iNKT cells can be extended against CD1d-negative tumor cells by their coating with αGC/CD1d-antitumor scFv fusion proteins. Two human tumor cell lines were selected based on their expression of HER2 and/or CEA (Fig. 3a). The pancreatic tumor cell line KATO III expresses both HER2 and CEA, as shown by the binding of the specific antibodies, as well as of the corresponding sCD1d-anti-HER2 and the newly developed sCD1d-anti-CEA fusion proteins (Fig. S1). In contrast, the breast cancer cell line SKBR-3 highly expresses HER2 but is negative for CEA, which provided the possibility of evaluating targeted versus untargeted iNKT cell-mediated cytotoxicity. After 4-h incubation with iNKT cells, tumor cells were killed only when coated with the relevant sCD1d-scFv fusion proteins. Indeed, KATO III tumor cells co-expressing HER2 and CEA were killed in the presence of either of the two αGC/sCD1d-antitumor proteins (Fig. 3b, left panel), with 40 and 60 % of tumor cells killed at an E/T ratio of 30/1 and 10 μg/ml of αGC/sCD1d-anti-HER2 and αGC/sCD1d-anti-CEA fusion proteins, respectively. In contrast, the SKBR-3 tumor cells expressing only HER2 were exclusively killed when incubated with the αGC/sCD1d-anti-HER2 protein (Fig. 3b, right panel), with 80 % of cells eliminated, while co-incubation with the αGC/sCD1d-anti-CEA protein resulted in only background ^51^Cr release similar to medium alone. At an E/T ratio of 10/1 (Fig. 3c), 20 % targeted killing of KATO III (left panel) and 50 % killing of SKBR-3 (right panel) tumor cells was still obtained with 1 μg/ml (13 nM) of HER2 and/or CEA-targeted CD1d fusion proteins, demonstrating the sensitivity of this approach. All iNKT cell clones tested in cytotoxic assays were CD4^+^ or DN and showed similar capacity of tumor-targeted cell killing.

### Activated iNKT cells exhibit poor bystander cytotoxicity and selectively kill CD1d-positive target cells

**Fig. 4:**
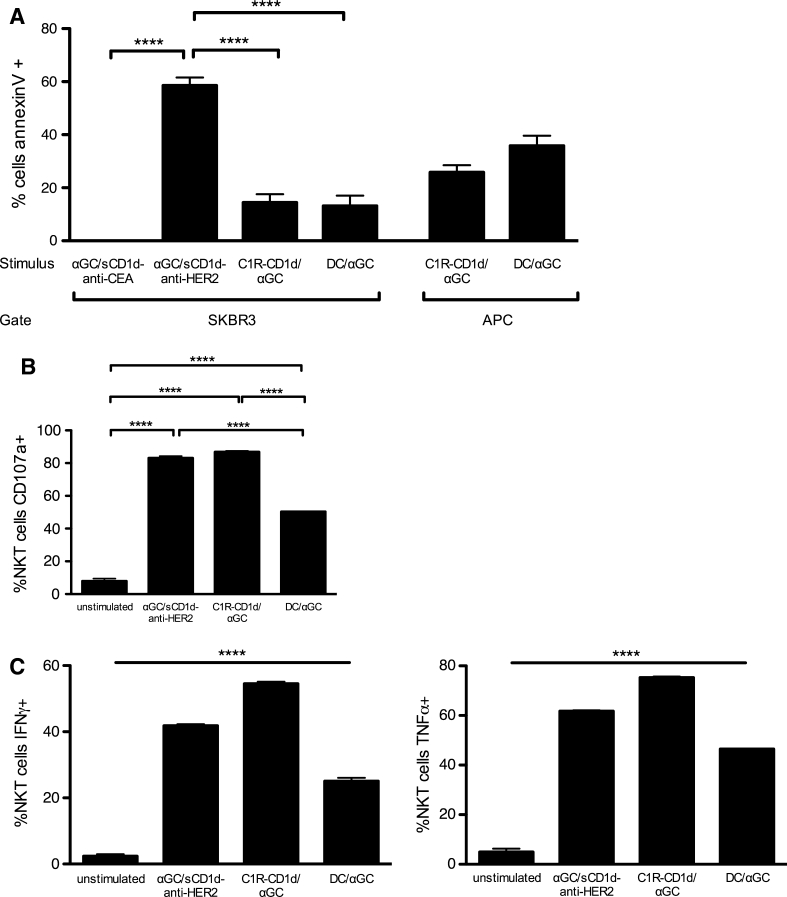
Activated iNKT cells kill αGC/sCD1d-loaded tumor cells as well as CD1d-positive APCs. **a** Apoptosis of SKBR3 tumor cells or APCs after 4-h incubation with iNKT cell clones, in the presence of αGC/sCD1d-CEA, αGC/sCD1d-HER2, αGC-pulsed C1R-CD1d cells, or αGC-pulsed moDCs. Results are shown as percentages of Annexin V^+^ 7AAD^−^ cells gated on HER2^+^ SKBR-3 cells, CD20^+^ C1R-CD1d, or CD11c^+^ moDCs, after subtracting the respective backgrounds obtained with unstimulated iNKT cells. **P < 0.005. **b** Cytotoxic activity of iNKT cells in the same experiment described in **a** was determined by assessing CD107a expression on the CD3^+^CD1d-tetramer^+^ effector cells. ***P < 0.001. **c** The activation state of iNKT cells was also evaluated by ICS for IFNγ and TNFα. The unstimulated group consists of the pooled data of iNKT cells incubated with unpulsed C1R-CD1d cells or moDCs. ****P < 0.0001 between all groups

In addition to ^51^Cr release experiments, the killing of SKBR3 tumor cells was evidenced by their Annexin V^+^ 7-AAD^−^ profile, while the state of iNKT cell activation was evaluated by CD107a expression, and secretion of TNFα and IFNγ (Fig. 4). After 4-h incubation with human iNKT cells, an average of 60 % of SKBR3 tumor cells was apoptotic when incubated with the tumor-targeted αGC/sCD1d-anti-HER2 fusion protein, while there was a similar background of tumor cell death with the untargeted αGC/sCD1d-anti-CEA or with unstimulated iNKT cells (Fig. 4a). Interestingly, when instead of recombinant CD1d proteins, iNKT cells and SKBR-3 tumor cells were co-incubated with αGC-pulsed C1R-CD1d cells or moDCs, less than 20 % of SKBR3 cells became Annexin V positive (Fig. 4a), although the percentages of CD107a, IFNγ and TNFα-positive iNKT cells (Fig. 4b, c) were similar after incubation with the tumor-targeted αGC/sCD1d-anti-HER2 protein or with αGC/C1R-CD1d cells. The iNKT cell activation was slightly weaker in the presence of αGC/moDCs (Fig. 4b, c), likely resulting from their hundred times lower CD1d expression than the C1R-CD1d transfectant. In addition, the state of activation of iNKT cells incubated with the αGC/sCD1d-anti-CEA did not differ from unstimulated iNKT cells (Fig. S2). In conclusion, the similar percentages of activated iNKT cells stimulated by αGC-pulsed APCs or by tumor-targeted αGC/sCD1d-anti-HER2 protein did not correlate with a similar killing of SKBR3 tumor cells, but rather with the concomitant elimination of the αGC/APCs, as shown by, respectively, 26 and 36 % of Annexin V^+^ αGC/C1R-CD1d and αGC/moDCs (Fig. 4a). Altogether, these data further confirmed the requirement of CD1d on the surface of the target cell for efficient killing, whether naturally expressed on the surface or bound via its fusion to an antitumor scFv fragment. The activation of iNKT cells by tumor-targeted CD1d molecules was also evidenced by their cytokine content (Fig. 4c). Indeed, in the presence of SKBR3 tumor cells coated with αGC/sCD1d-anti-HER2, about half of iNKT cells was positive for TNFα and IFNγ, while no intracellular cytokines were detected in the presence of the irrelevant αGC/sCD1d-anti-CEA fusion protein. Altogether, these results support the relevance of sCD1d-antitumor fusion proteins for cancer therapy, as seen by the strong activation of human iNKT cells, revealed by direct tumor cytotoxicity and cytokine release.

### In vivo targeting of αGC-loaded CD1d proteins to the tumor site is required for prolonged iNKT cell-mediated tumor inhibition

**Fig. 5:**
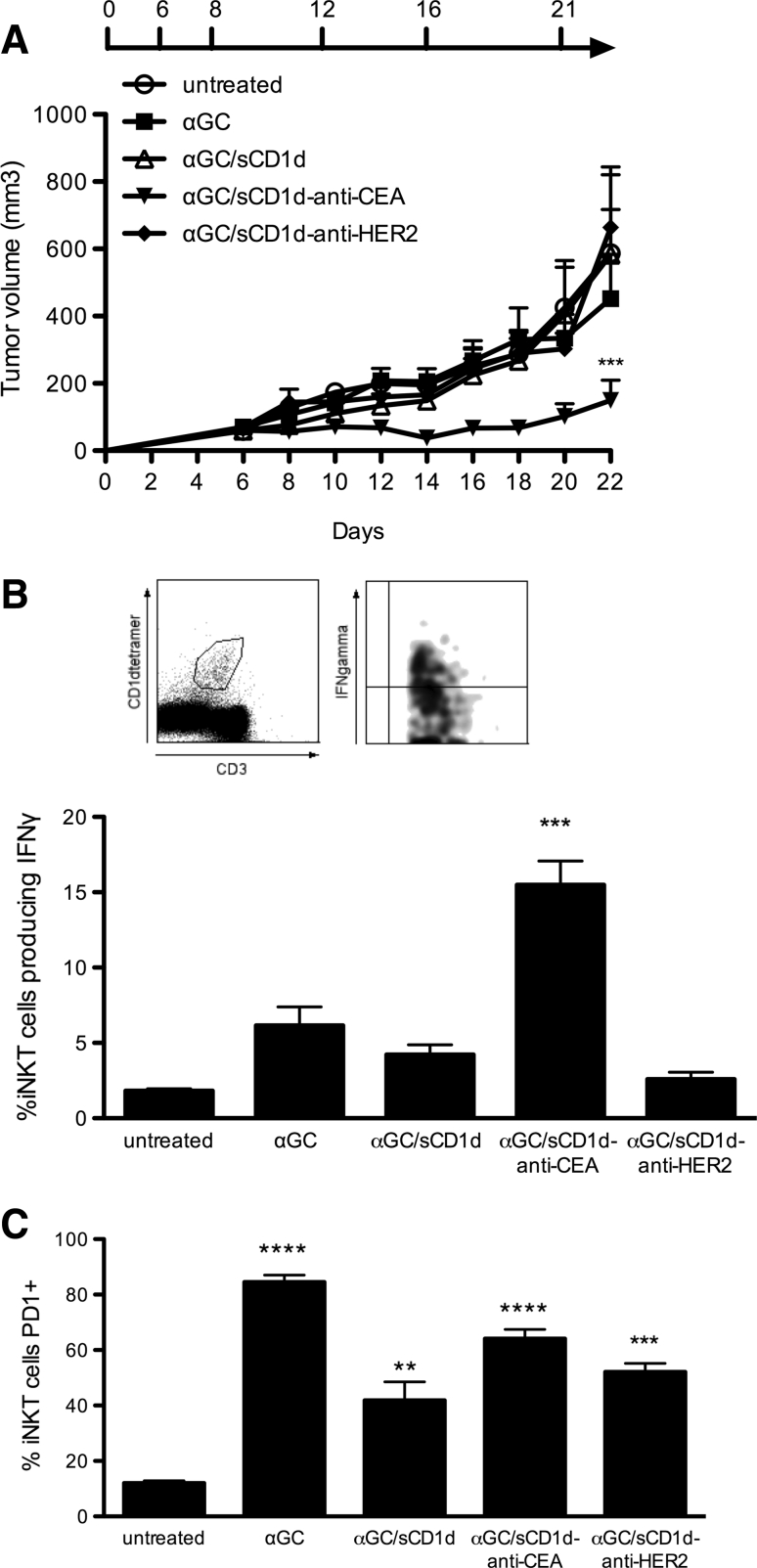
In vivo antitumor activity of αGC/sCD1d-anti-CEA fusion protein. **a** Mice were grafted s.c. with 7 × 10^5^ MC38-CEA tumor cells. I.v. injections of PBS (untreated), or equimolar amounts of αGC, αGC-loaded sCD1d, αGC/sCD1d-anti-HER2, and αGC/sCD1d-anti-CEA were started 6 days later when all tumors were well established and were repeated for a total of 5 injections as specified. Tumors were measured every 2 days and the graph represents the kinetic of tumor growth (mm^3^) as the mean of 7 mice per group. ***P < 0.001 versus untreated group. **b** Ex vivo IFNγ production by spleen iNKT cells at the end of the antitumor experiment (day 22). Splenocytes were isolated 1 h after the sixth injection of each treatment and iNKT cells were stained with CD1d-tetramer-PE and anti-CD3-FITC, and intracellularly with anti-IFNγ-APC. Results are expressed as the mean percentage of IFNγ-producing iNKT of three mice per group. ***P < 0.001 versus all groups. **c** The same samples as in **b** were analyzed for the expression of the co-inhibitory receptor PD-1 on spleen iNKT cells. The* graph* represents the percentage of PD-1 positive iNKT cells from three mice per group. **P < 0.005, ***P < 0.001, ****P < 0.0001 versus untreated group

The importance of targeting αGC/sCD1d fusion proteins to the tumor was further investigated in C57BL/6 mice grafted with the MC38 colon carcinoma cell line stably transfected with human CEA (MC38-CEA). Mice with established tumors (>100 mm^3^) were treated either with αGC/sCD1d-anti-CEA fusion protein (Fig. S1), αGC alone, untargeted αGC/sCD1d or αGC/sCD1d-anti-HER2 fusion proteins. After a total of six injections given over 3 weeks, all mice treated with αGC/sCD1d-anti-CEA protein retained small tumors barely exceeding 200 mm^3^, and hence 60 % smaller than in untreated animals (700 mm^3^) (Fig. 5a). In marked contrast, αGC alone and untargeted sCD1d were unable to inhibit tumor growth. The requirement of tumor-targeted CD1d treatment to achieve a therapeutic effect was best demonstrated in the mice treated with the irrelevant αGC/sCD1d-anti-HER2 fusion protein, as all animals had fast tumor growth. The prolonged reactivity of iNKT cells was tested in the spleen by the detection of ex vivo IFNγ production 1 h after the sixth injection. After repeated injections of αGC, only few iNKT cells still produced IFNγ, confirming the induction of anergy upon repetitive stimulations (Fig. 5b) [18, 19]. Similarly, no significant IFNγ was detected after untargeted αGC/sCD1d and irrelevant αGC/sCD1d-anti-HER2 treatments, though in this case likely resulting from a weak activation of iNKT cells rather than anergy. In contrast, 15 % of spleen iNKT cells isolated from mice treated with tumor-targeted αGC/sCD1d-anti-CEA were positive for IFNγ, which correlated with the fact that antitumor activity was exclusively obtained in this group. Although tumor-infiltrating iNKT cells were too few to be functionally analyzed, the prolonged reactivity of iNKT cells only in the spleens of mice treated with tumor-targeted fusion protein suggested that these cells had been repeatedly activated at the tumor site. In view of previous studies that attributed αGC-induced iNKT cell anergy to the up-regulation of the co-inhibitory receptor programmed death-1 (PD-1) [28, 29], we tested its expression level on spleen iNKT cells (Fig. 5c). Strikingly, PD-1 expression was up-regulated not only on the vast majority of iNKT cells after six injections of αGC alone, but also after repeated injections of recombinant αGC/sCD1d proteins, including iNKT cells activated by the tumor-targeted αGC/sCD1d-anti-CEA protein (Fig. 5b). Therefore, increased PD-1 expression did not correlate with the state of unresponsiveness of iNKT cells. At this point, it is interesting to mention that the majority of human iNKT cells were found positive for PD-1 expression when analyzed ex vivo in normal donor PBMCs (Fig. S3), likely resulting from previous in vivo stimulations. These observations suggest that PD-1 up-regulation on mouse and human iNKT cells is rather a marker of activation and is not in itself sufficient to mediate iNKT cell anergy.

### The prolonged reactivity of iNKT and NK cells to repeated stimulations is optimized by tumor bound CD1d proteins

**Fig. 6:**
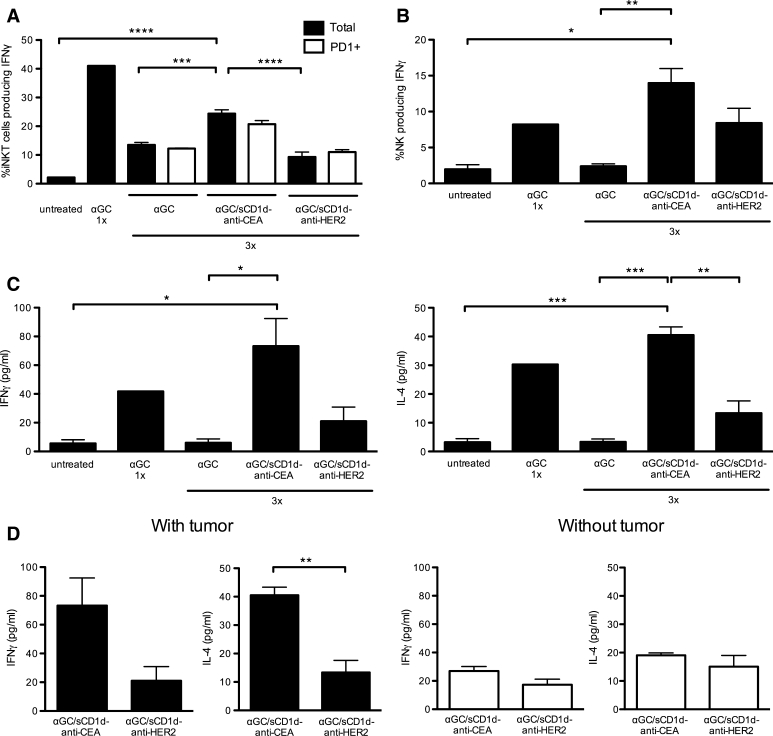
Sustained activation of iNKT and NK cells upon repeated injections of tumor-targeted αGC/sCD1d-anti-CEA. Mice bearing MC38-CEA tumors were treated three times, following the same protocol as in the tumor therapy experiments (Fig. 5), and splenocytes were analyzed 1 h after the third injection. As a positive control for IFNγ production, a naïve mouse was injected only once with αGC. **a** Percentage of IFNγ-producing cells gated on total (*black bars*) or PD-1^+^ (*empty bars*) iNKT cells. **b** Percentage of IFNγ-producing NK cells as gated on CD3^−^NK1.1^+^. **c** Cytokines were measured by CBA in the sera of mice taken 1 h after the third treatments, as described in **a**.* Graphs* show the concentration of IFNγ (*left panel*) and IL-4 (*right panel*). **d** Serum levels of IFNγ and IL-4 were compared between mice-bearing or not MC38-CEA tumors, and treated with targeted or non-targeted αGC/sCD1d fusion proteins administered as in **a**. All data are shown as the mean ± SEM of 3 mice per group, *P < 0.01, **P < 0.005, ***P < 0.001, ****P < 0.0001

The requirement for CD1d tumor targeting to provide sustained reactivity of iNKT despite PD-1 up-regulation was further characterized. As already reported [18, 19], a single injection of αGC induced a fast and potent activation of iNKT cells, as revealed by the presence of 40 % splenic iNKT cells producing IFNγ (Fig. 6a). However, after three injections of αGC and despite similar numbers of iNKT cells (data not shown), only 12 % of iNKT cells still produced IFNγ (Fig. 6a), confirming the induction of anergy upon repetitive stimulations [18, 19]. In contrast, 25 % of iNKT cells from MC-38-CEA tumor-bearing mice treated with αGC/sCD1d-anti-CEA fusion protein still produced IFNγ (Fig. 6a), while only 9 % iNKT cells were IFNγ^+^ in mice treated with the irrelevant αGC/sCD1d-anti-HER2 fusion protein. Importantly, after three treatments, percentages of IFNγ-producing cells were similar whether gated on total or on PD-1^+^ iNKT cells (Fig. 6a), confirming that PD-1 up-regulation was not sufficient to block the restimulation of iNKT cells. In view of their fast transactivation by iNKT cells, NK cells were tested in the same groups of mice (Fig. 6b). After three injections of αGC, spleen NK cells also failed to produce IFNγ, as a consequence of iNKT cell anergy. In contrast, in mice treated with either αGC/sCD1d-anti-CEA or αGC/sCD1d-anti-HER2 fusion proteins, NK cells remained reactive, as seen by increased IFNγ production. The repeated activation of NK cells upon treatment with the irrelevant fusion protein αGC/sCD1d-anti-HER2 indicated that sustained systemic activation of iNKT cells had occurred, although to a weaker extent than with the tumor-targeted treatment. Serum cytokines measured 1 h after the third injection also reflected the prolonged reactivity of iNKT cells to tumor-targeted recombinant CD1d proteins (Fig. 6c), as shown by significant serum levels of IFNγ and IL-4 in mice treated with αGC/sCD1d-anti-CEA protein, while barely any cytokines could be measured after three injections of αGC/sCD1d-anti-HER2 protein. Regarding αGC as a free drug, a single injection induced a fast release of IFNγ and IL-4, but almost no cytokine production was detected after three injections, confirming the induction of iNKT cell anergy. Most importantly, in the absence of MC38-CEA tumor grafts, repeated treatments with αGC/sCD1d-anti-CEA fusion protein did not lead to significant release of cytokines, which did not differ from mice treated with the irrelevant αGC/sCD1d-anti-HER2 protein (Fig. 6d). Altogether, these results demonstrate the importance of targeting CD1d to the tumor site to favor a strong and prolonged reactivity of iNKT cells.

## Discussion

The present study demonstrates the therapeutic efficacy of recombinant sCD1d-antitumor scFv fusion proteins via the sustained activation of murine and human cytolytic iNKT cells. The attractiveness of this strategy resides in two main beneficial characteristics. First, recombinant CD1d proteins have the capacity to keep iNKT cells reactive through multiple stimulations, in contrast to their unresponsiveness after repeated challenge by αGC-loaded APCs. Second, the targeting of CD1d molecules to cancer cells by their fusion to an antitumor scFv fragment efficiently redirects activated iNKT cells to the tumor site, promoting a local innate immune response, including direct lysis of targeted tumors by iNKT cells, release of large amounts of cytokines and transactivation of NK cells, altogether leading to prolonged antitumor effects.

So far, the therapeutic use of iNKT cells has been limited by their short-lived cytokine response to αGC stimulation, followed by a long-term anergy [4, 17, 18]. Several mechanisms have been proposed for the induction of iNKT cell unresponsiveness, and the controversial results suggest that multiple factors are likely involved. At first, the fast and long-term up-regulation of PD-1 upon activation of iNKT cells was proposed as the main mechanism since their anergic state could be prevented or reverted by PD-1/PD-L1 blockade and did not occur in PD-1 KO mice [[28], [29]]. However, the exclusive role of PD-1 in the control of iNKT cell anergy was not confirmed in all systems [30], and the involvement of co-stimulation through CD28 or co-inhibitory receptors such as BTLA was suggested [30–32]. In this respect, our present results also demonstrated the strong and long-term up-regulation of PD-1 upon activation of murine iNKT cells, which however did not correlate with the state of anergy. Indeed, iNKT cells remained reactive to multiple injections of recombinant CD1d fusion proteins despite similar up-regulation of PD-1 as was found on anergic iNKT cells after treatment with αGC as a free drug. Clinical trials in cancer patients have preferred the autologous transfer of αGC-pulsed DC, which showed prolonged iNKT cell activation, as compared to the glycolipid alone [4, 11, 13, 14, 33]. However, a maximum of two subsequent rounds of iNKT cell stimulations were seen despite three to four autologous transfers of αGC-pulsed DC in some studies, suggesting that iNKT cells became progressively unresponsive to further challenge. Unfortunately, in vitro experiments with human iNKT cells do not allow monitoring the induction of anergy by αGC, likely due to the presence of IL-2 required for the maintenance of human and mouse iNKT cells in vitro. Indeed, IL-2 has been shown to prevent or revert the state of anergy of murine iNKT cells both in vivo and in vitro [18, 34]. However, it is interesting to note that around 70 % of human iNKT cells were already expressing PD-1 when tested ex vivo on total PBMC from healthy donors, probably resulting from previous in vivo antigen stimulations. Since it is unlikely that the large majority of iNKT cells present in normal donors are anergic, the expression of PD-1 on activated human and mouse iNKT cells is probably not sufficient to mediate iNKT cell unresponsiveness. So far, the exact mechanism that mediates the αGC-induced anergy of iNKT cells or, conversely, their prolonged reactivity to recombinant αGC/CD1d fusion proteins remains unclear.

Importantly, our data demonstrated a direct activation of human iNKT cells by recombinant soluble CD1d proteins in the absence of any APC, which excluded the possibility of loss of αGC from the recombinant CD1d proteins and its loading onto endogenous CD1d expressed by APCs. In this regard, the capacity of a monomeric CD1d molecule to activate iNKT cells seems to be unique to this antigen-presenting molecule, as soluble MHC Class I and Class II monomers were shown to be unable to activate antigen-specific T cells, in contrast to dimeric and multimeric forms [35, 36]. The question remains whether the iNKT cell activation by monomeric αGC/sCD1d proteins is peculiar to the strong agonist αGC and whether it would not occur with a more physiological CD1d ligand. Nevertheless, activation of human iNKT cells by soluble αGC/CD1d proteins remained significantly less efficient than plastic-coated or tumor bound CD1d fusion proteins and αGC-pulsed APCs, which likely resulted from the lack of molecular CD1d aggregation in the absence of plastic or cell surface, as well as from the absence of co-stimulation. In support of this, systemic treatments of mice with untargeted αGC/sCD1d or irrelevant αGC/sCD1d-scFv fusion proteins led to a two to threefold weaker release of cytokines, as compared to treatment with the tumor-targeted αGC/sCD1d-scFv protein. Rather than a drawback, the lower potency of soluble monomeric CD1d molecules will be rather favorable in limiting a potentially detrimental systemic activation of iNKT cells, while promoting their sustained activation when targeted at the tumor site.

The second attractive characteristic of our immunotherapy strategy is the targeting of recombinant CD1d fusion proteins to tumors by fusion of the extracellular part of CD1d to an antibody scFv fragment specific for a tumor antigen. Indeed, the present data show both in vitro with human iNKT cell clones and in vivo in a therapeutic mouse tumor model that the strongest tumor inhibition is obtained with tumor-targeted recombinant sCD1d-antitumor scFv fusion protein as compared to an irrelevant sCD1d fusion protein. This finding fits with the prerequisite of CD1d expression by tumors to promote efficient iNKT cell-mediated killing, which has been previously reported in humans in the context of CD1d-expressing lymphomas [26, 37, 38], and in mice with tumor models transfected with CD1d [39, 40]. In this context, our strategy using CD1d-antitumor scFv fusion proteins opens the possibility to target CD1d-negative tumors and render them susceptible to iNKT cell attack. The effectiveness of this approach was best demonstrated by the direct cytotoxicity of human iNKT cell clones against tumor cells when coated with the αGC/sCD1d-scFv fusion protein specific for the tumor antigen expressed on their surface, while the untargeted αGC/sCD1d-scFv was unable to induce antitumor cytotoxic activity. More importantly, although iNKT cells were strongly activated by αGC-pulsed APCs, such as C1R-CD1d or moDCs, as seen by CD107a expression and cytokine release, they rather killed the APCs and to a lesser extent the tumor cells, indicating that bystander killing by iNKT cells was inferior to the CD1d-mediated cytotoxicity. These observations suggest that the strategy of iNKT cell activation by autologous transfer of αGC-pulsed DCs, as tested so far in clinical trials, may direct the intrinsic cytotoxic activity of iNKT cells preferentially against the transferred DCs and not against tumors. Because of the relatively low numbers of peripheral iNKT cells in humans, this aspect was probably not a major issue, and these protocols of DC transfer have instead successfully induced the immunomodulatory functions of iNKT cells, such as the transactivation of NK and T cells. However, our results in mice demonstrated that the stronger iNKT cell activation by tumor-targeted sCD1d treatment also led to higher NK cell activation, which are known to greatly participate in tumor inhibition [19]. Therefore, the targeting of CD1d molecules to the tumor site not only triggers iNKT-mediated tumor lysis, but also favors local innate and adaptive antitumor responses, as suggested by the accumulation of iNKT, NK, and T cells at the tumor site [19]. The importance of tumor targeting and local activation of iNKT cells was underlined by recent clinical trials in which αGC-pulsed DC and/or ex vivo expanded iNKT cells were delivered in the vicinity of the tumor [15, 33]. The best clinical results were obtained in HNSCC patients in whom αGC-pulsed DCs were administered via the nasal submucosa and iNKT cells via the tumor-feeding arteries [15, 41]. In addition to the local delivery of iNKT cells, this encouraging clinical benefit also demonstrated the need to increase the numbers of iNKT cells by autologous transfer of in vitro expanded iNKT cell lines, since the frequency of iNKT cells is often very low or even undetectable in advanced cancer patients [8, 42]. To this aim, recombinant sCD1d-antitumor fusion proteins appear as promising tools: first, in vitro to expand large numbers of human iNKT cells for adoptive transfer, and second, in vivo to redirect these transferred iNKT cells to the tumor site.

Finally, the efficient tumor targeting of CD1d molecules requires the over-expression of a tumor antigen for which a high-affinity antibody scFv has been developed. So far, we have used two of the highest affinity existing scFv fragments specific for the HER2 and CEA, which are often over-expressed, respectively, in breast cancers and gastric cancers. As alternatives to tumor-associated markers, antigens over-expressed in the tumor stroma and/or neo-vessels would be additional good candidates for the targeting of CD1d molecules to the tumor site.

In conclusion, the present results propose and support monomeric CD1d-scFv antitumor fusion proteins as a potent tool to effectively harness iNKT cells against cancer.

## Electronic supplementary material

Below is the link to the electronic supplementary material.

Supplementary material 1 (PDF 337 kb)
