# Supplementary material for: CD1d-antibody fusion proteins target iNKT cells to the tumor and trigger long-term therapeutic responses
Source: Cancer Immunol Immunother. 2012 Dec 15;62(4):747–60. doi: 10.1007/s00262-012-1381-7 (PMC3624007; doi:10.1007/s00262-012-1381-7)
Supplement: Supplementary file 1 — Supplementary material 1 (PDF 337 kb) [file 262_2012_1381_MOESM1_ESM.pdf]

Supplementary figure 1

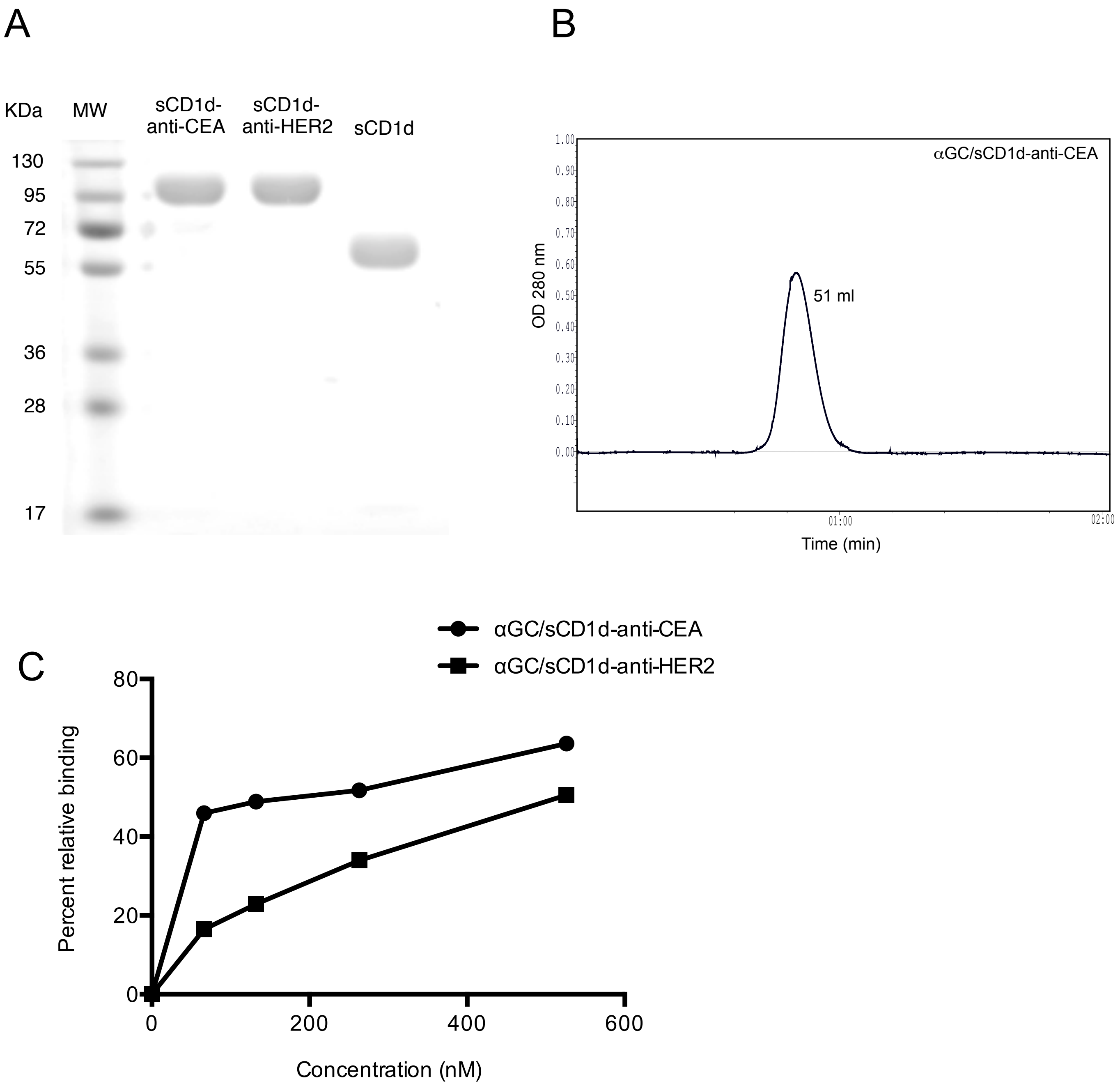

Supplementary Fig 1. Characterization of the CD1d-anti-CEA fusion protein. **A** The purities of sCD1d fusion proteins are shown in a 10% SDS-PAGE after purification by Ni-NTA chromatography and **B** FPLC Sephacryl S100 profile after loading with αGC. **C** Titration of the binding of the sCD1d–anti-HER2 or sCD1d–anti-CEA on B16-HER2 or B16-CEA cells. Data are displayed as the relative percentage of the binding of the respective full anti-HER2 Herceptin or anti-CEA antibodies. Detection was performed using anti-CD1d–FITC and anti-human IgG FITC, respectively. For detail methods see Stirnemann et al, JCI, 2008 (Reference 19).

Supplementary figure 2

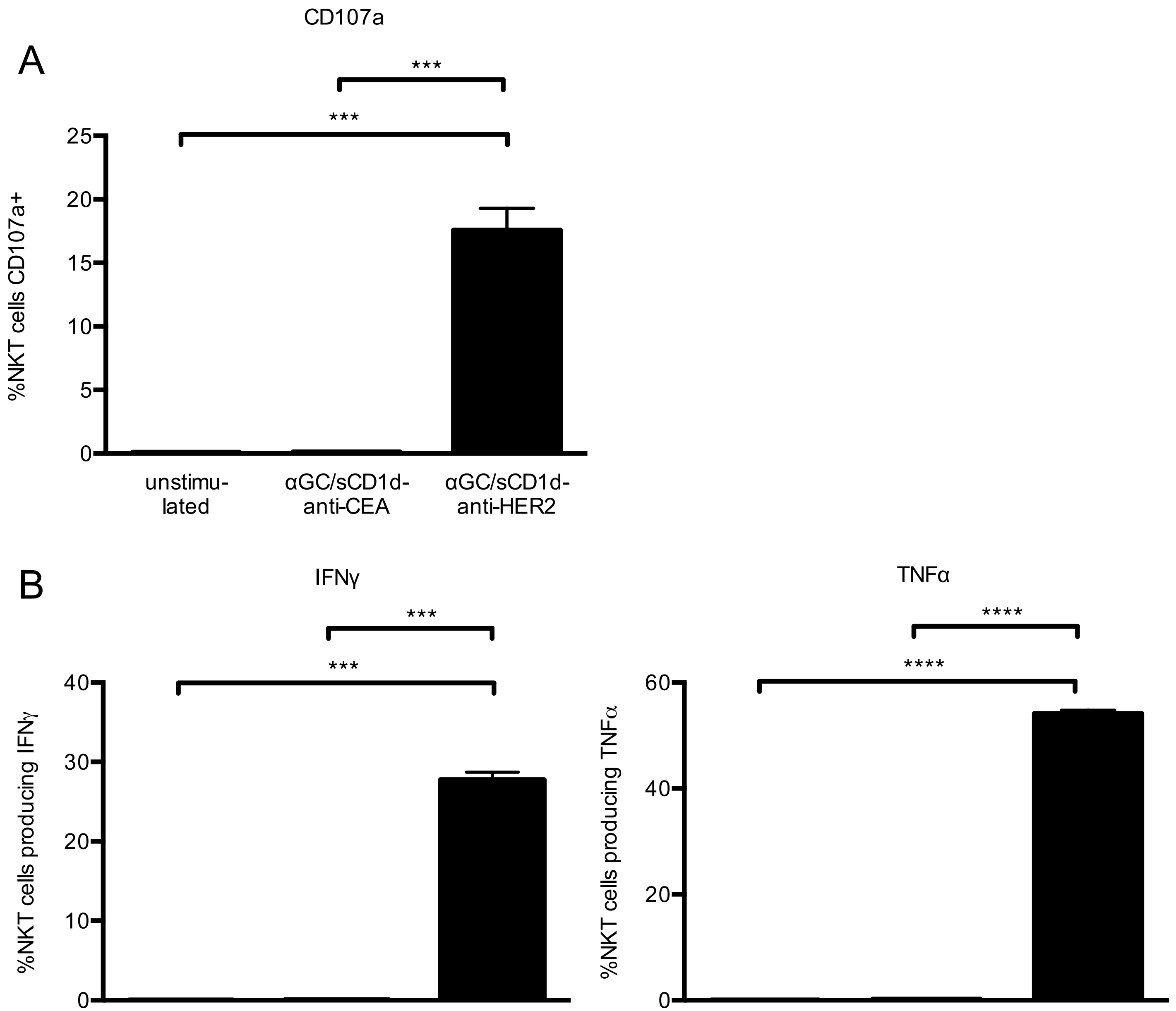

Supplementary Fig 2. The activation state of iNKT cells with either targeted or non-targeted fusion proteins. iNKT cell clones were incubated for four hours with SKBR3 tumor cells alone or in the presence of αGC/sCD1d-CEA or αGC/sCD1d-HER2. Results are shown as percentages of $CD3^{+}$CD1d-tetramer^{+} effector cells expressing CD107a **(A),** IFNγ and TNFα **(B).** ***P<0.001, ****P<0.0001. A detail description can be found in the materiels and methods section.

Supplementary figure 3

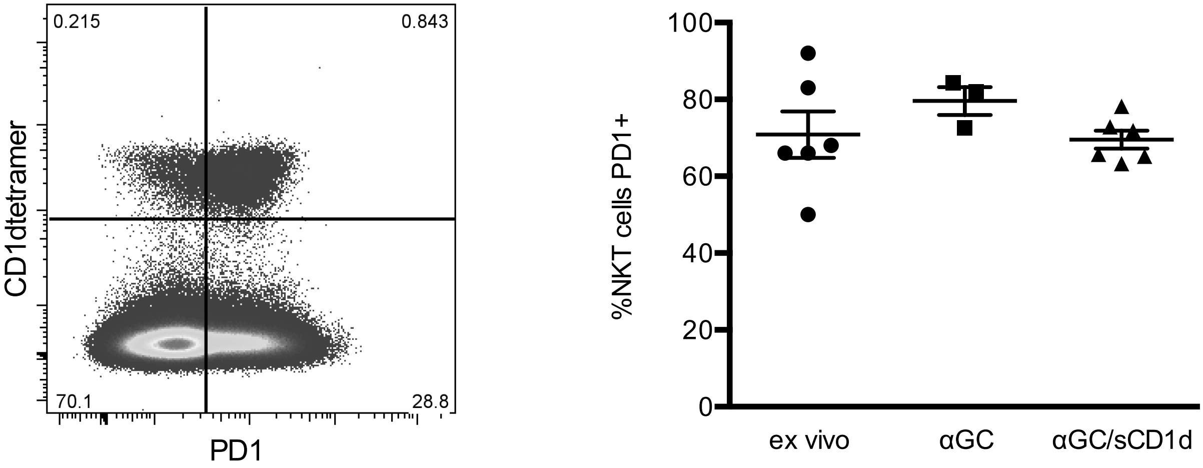

Supplementary Fig 3. Expression of PD1 on human iNKT cell lines. Human PBMCs were stimulated as described in Fig 1. iNKT cells were labeled with human anti-PD1 antibody and analyzed with a LSRII (BD Biosciences) and the acquired data were processed using FlowJo software (Tree Star Inc.).
